# Novel proteins associated with chronic intermittent hypoxia and obstructive sleep apnea: From rat model to clinical evidence

**DOI:** 10.1371/journal.pone.0253943

**Published:** 2021-06-29

**Authors:** Xiaojun Tang, Shisheng Li, Xinming Yang, Qinglai Tang, Ying Zhang, Shiying Zeng, Mengmeng Li, Kang Jiang, Lu Guo, Peiying Huang

**Affiliations:** Department of Otolaryngology Head and Neck Surgery, The Second Xiangya Hospital, Central South University, Changsha, Hunan, China; Sapienza University of Rome, ITALY

## Abstract

**Objective:**

To screen for obstructive sleep apnea (OSA) biomarkers, isobaric tags for relative and absolute quantitation (iTRAQ)-labeled quantitative proteomics assay was used to identify differentially expressed proteins (DEPs) during chronic intermittent hypoxia (CIH).

**Method:**

The iTRAQ technique was applied to compare DEPs in the serum of a CIH rat model and control group. Biological analysis of DEPs was performed using Gene Ontology and Kyoto Encyclopedia to explore related biological functions and signaling pathways. Enzyme-linked immunosorbent assay (ELISA) was performed to validate their expression in sera from patients with OSA and CIH rats.

**Results:**

Twenty-three DEPs (fold change ≥1.2 or ≤0.833, *p*<0.05) were identified, and two DEPs (unique peptides>3 and higher coverage) were further verified by ELISA in the CIH rat model and OSA subject: apolipoprotein A-IV (APOA4, *p*<0.05) and Tubulin alpha-1A chain (TUBA1A, *p*<0.05). Both groups showed significant differences in the expression levels of DEPs between the CIH and control groups and the severe OSA and non-OSA groups. APOA4 was found to be upregulated and TUBA1A downregulated in both the sera from OSA patients and CIH rats, on comparing proteomics results with clinical results. There were two pathways that involved three DEPs, the mitogen-activated protein kinase (MAPK) signaling pathway (*p*<0.05) and cytokine-cytokine receptor interaction (*p*<0.05).

**Conclusion:**

APOA4 and TUBA1A may be potential novel biomarkers for CIH and OSA, and may play an important role in the development of OSA complications.

## Introduction

Obstructive sleep apnea (OSA), a common respiratory disorder characterized by recurring conditions of complete or partial collapse of the upper airway during sleep, is an independent risk factor for many clinical complications [1], such as cardiovascular and olfactory pulmonary comorbidities, the severity of which is greater in the elderly [2,3]. The estimated prevalence of OSA in the general population is 3–6% and is higher in middle-aged or elderly people [4]. Apnea or hypopnea results from upper airway obstruction, leading to chronic intermittent hypoxia (CIH), carbon dioxide retention, repeated intrathoracic negative pressure, and abnormal sleep structure, which are linked to oxidative stress, inflammation, and cardiovascular and metabolic diseases. Conversely, cardiovascular risk in patients with OSA is reduced after non-resectable pharyngoplasty because apnea or hypopnea improves after treatment [5].

It is well understood that CIH is a main feature of OSA and leads to related complications. CIH increases the production of reactive oxygen and enhances the level of oxidative stress, promoting sympathetic excitement and blood pressure, contributing to endothelial dysfunction, and thus predisposing patients to complications, such as cardiovascular disease, metabolic dysfunction, and cognitive decline [6]. However, the specific contribution of CIH to OSA complications remains unclear.

There have been several recent proteomics [7–10] and genomics [11–15] studies aimed at elucidating the pathophysiology of OSA. Isobaric tags for relative and absolute quantitation (iTRAQ) have been widely employed in quantitative proteomics, and many recent studies have focused on OSA [10] and CIH. Even though selected proteins are involved in OSA-related pathways, the effect of OSA, especially CIH, on proteomics and genomics remains largely unknown. In addition, research often presents with confounding factors that cannot completely rule out whether the subjects have other OSA-induced diseases. To overcome these drawbacks, animal models of OSA are periodically exposed to normoxia/hypoxia to simulate apnea/hypopnea in patients with OSA. Studying the specific pathophysiology of CIH in OSA and the exact underlying mechanisms is critical.

In this study, we performed proteomics analysis of serum obtained from a CIH rat model to identify differentially expressed proteins (DEPs) by iTRAQ. Bioinformatic analyses were conducted to reveal the biological characteristics and potential pathways of DEPs, which were further verified in a CIH rat model and in humans. Overall, these findings may provide a strong foundation for further research on the role of CIH in OSA.

## Methods

### Serum samples collection

The experiments were performed on male Sprague-Dawley rats (170–200 g, n = 6) obtained from Hunan SJA Laboratory Animal Co. Ltd.. Rats were divided into two groups: normoxia (21% O_2_, 24 h per day, 9 weeks, n  =  3) and CIH group (5% O_2_ for 60 s, 21% O_2_ for 120 s, 8 h per day, 9 weeks, n  =  3). For CIH exposure, rats were placed in a polymethyl methacrylate hypoxic chamber that was connected to a supply of N_2_ and O_2_ gas. The chamber was periodically filled with N_2_ and O_2_ to regulate the degree and time of hypoxia and was equipped with sensors to monitor the O_2_ concentration. The rats had free access to water and chow with consistent temperature, humidity, and light-dark cycle (light from 07:00 to 19:00). After exposure, the chamber was cleaned to discharge the residual gas and normalize other factors affecting the experimental treatment.

After a 9-week intervention and exposure to respective treatment, rats were anesthetized and serum samples were collected. Serum samples (approximately 3 mL) were collected from the antecubital inferior caval vein, allowed to clot for 30 min, and centrifuged at 3000 *g* for 10 min at 25°C. The resulting serum was aliquoted and frozen at 80°C for further analysis.

All experimental procedures were approved by the Second Xiangya Hospital of Central South University and were in accordance with the laboratory guidelines.

### High-abundance proteins removal

Using Agilent Multiple Affinity Removal LC Column Mouse-3 to remove high-abundance proteins, a low-abundance component solution was prepared. Ultrafiltration concentration was carried out with a 10 kD ultrafiltration tube, and one volume of SDT pyrolysis (4% SDS, 100 mM Tris-HCl, pH 7.6) was added. The solution was placed in boiling water for 15 min and centrifuged at 14000 *g* for 15 min. The supernatant was collected, and the protein concentration was measured using a commercial assay that relies on the Bradford method (Beyotime, Shanghai, China).

### Protein digestion and iTRAQ labeling

Proteins were reduced, alkylated, and enzymatically digested overnight with trypsin. For labeling of iTRAQ, Reagent‐8plex Multiplex Kit (AB SCIEX) was used according to the manufacturer’s protocol. The iTRAQ labeling was performed twice: 113, 114, and 117 tags for CIH samples, and 118, 119, and 121 tags for control samples.

### Off-line two dimensional LC-MS/MS

The mixed peptides were fractionated by an Agilent 1260 Infinity II HPLC system and separated on a column (Thermo scientific, Acclaim PepMap RSLC 50 μm × 15 cm, nano viper, P/N164943) using an Easy nLC chromatographic system (Thermo Scientific). After separation by chromatography, the samples were analyzed using a Q-Exactive Plus mass spectrometer. The detection method used positive ions. The parent ion scanning range was–350–1800 m/z.

### Data analysis

Raw data and protein quantification were analyzed using Mascot (version 2.5, Matrix Science) and Proteome Discoverer (version 2.1, Thermo Fisher Scientific Inc. 2014). Briefly, a Proteome Discoverer was used to convert the original atlas file (.raw file) generated by the Q Exactive Plus.mgf file, which is uploaded to the Mascot server for database retrieval through the built-in software tools. The file then created on the Mascot server (.dat file) is transmitted back to the software through Proteome Discoverer, and the data are filtered according to the standard of false discovery rate <0.01 to obtain highly reliable qualitative results.

### Bioinformatic analysis

All identified peptides had an ion score above the Mascot peptide identity threshold (a high confidence score of 99% and a low false discovery rate of 1%), and a target protein was considered identified if at least two such unique peptide matches were apparent for the protein. We set a 1.2-fold up- or down-regulation change and *p* value (*t*-test) <0.05, as the threshold to identify significant changes. Gene Ontology (GO) functional classifications were analyzed with Blast2GO, and Kyoto Encyclopedia of Genes and Genomes (KEGG) pathway annotations were performed using KAAS (KEGG Automatic Annotation Server). GO and KEGG enrichment analyses were performed to identify GO terms that were significantly enriched in differentially expressed proteins by Fisher’s exact test.

### Animal model of CIH and validation

Another group of rats was divided into two groups: normoxia (21% O_2_, 24 h per day, 9 weeks, n  =  10) and CIH group (5% O_2_ for 60 s, 21% O_2_ for 120 s, 8 h per day, 9 weeks, n  =  10).

Serum samples were collected as described previously. Serum apolipoprotein A-IV (APOA4) and tubulin alpha-1A chain (TUBA1A) levels were measured in each serum sample. All ELISA tests were performed using commercially available kits (Bioswamp, Jiangsu, China) in duplicate following the manufacturers’ directions, and all samples were assayed with blinded sample characteristics.

### Recruitment of clinical subjects and human serum samples collection

The study was approved by the Medical Ethics Committee of the Second Xiangya Hospital. All participants signed informed consent forms. All researchers collected information blinds from individuals.

All 41 individuals, who were diagnosed with OSA by overnight polysomnography between September 2018 and June 2019 at the Second Xiangya Hospital of Central South University, were men and aged between 22 and 62 years, with clinical symptoms and signs compatible with clinical OSA. Exclusion criteria included central sleep apnea, cancer, autoimmune disease, chronic or acute infections, inflammatory illness, and autoimmune disease. In addition, subjects were excluded if they had NYHA grade IV heart failure, chronic renal failure (stage 4–5), degenerative cerebrovascular disease, or severe lung disease. None of the subjects were taking anti-inflammatory medications.

According to the apnea–hypopnea index (AHI), subjects were classified into non-OSAS (AHI <5, n = 12) and OSAS group (AHI≥5, n = 29). The OSAS group was assigned as follows: mild to moderate: AHI > 5 and ≤30 (n = 14); or severe, AHI >30 (n = 15).

Blood samples (3–5 ml) were collected by antecubital venipuncture, in accordance with the standard hospital extraction procedure. Samples were taken at 06:30 after overnight polysomnography was completed in a fasting state. The rest of the procedure for obtaining human serum samples was as mentioned above (serum samples collection section).

Serum IApolipoprotein A-IV (APOA4) and tubulin alpha-1A chain (TUBA1A) levels were measured in each serum sample of the validation set. All ELISA tests were performed in commercially available kits (Bioswamp, Jiangsu, China) in duplicate, following the manufacturers’ directions, and all samples were assayed with sample characteristics blinded.

### Statistical analysis of ELISA data

All analyses were conducted using SPSS software (version 24.0; SPPS Inc., Chicago, IL, USA), and all data are expressed as mean ± standard deviation. Men were subdivided into three groups based on OSA. Between-group comparisons of clinical continuous variables were performed using the Mann–Whitney U test, at a 95% confidence interval. Pearson correlation and linear regression analyses were conducted to examine potential associations between AHI, lowest oxygen saturation, and APOA4 and TUBA1A serum concentrations. All correlations were bilateral, and *p* <0.05 was considered significant.

## Results

### CIH caused a protein expressional profile

To identify differentially expressed proteins (DEPs) in the CIH process, protein expression profiles between the CIH rat model and controls were compared using iTRAQ Labeling and two dimensional LC-MS/MS.

**Fig 1:**
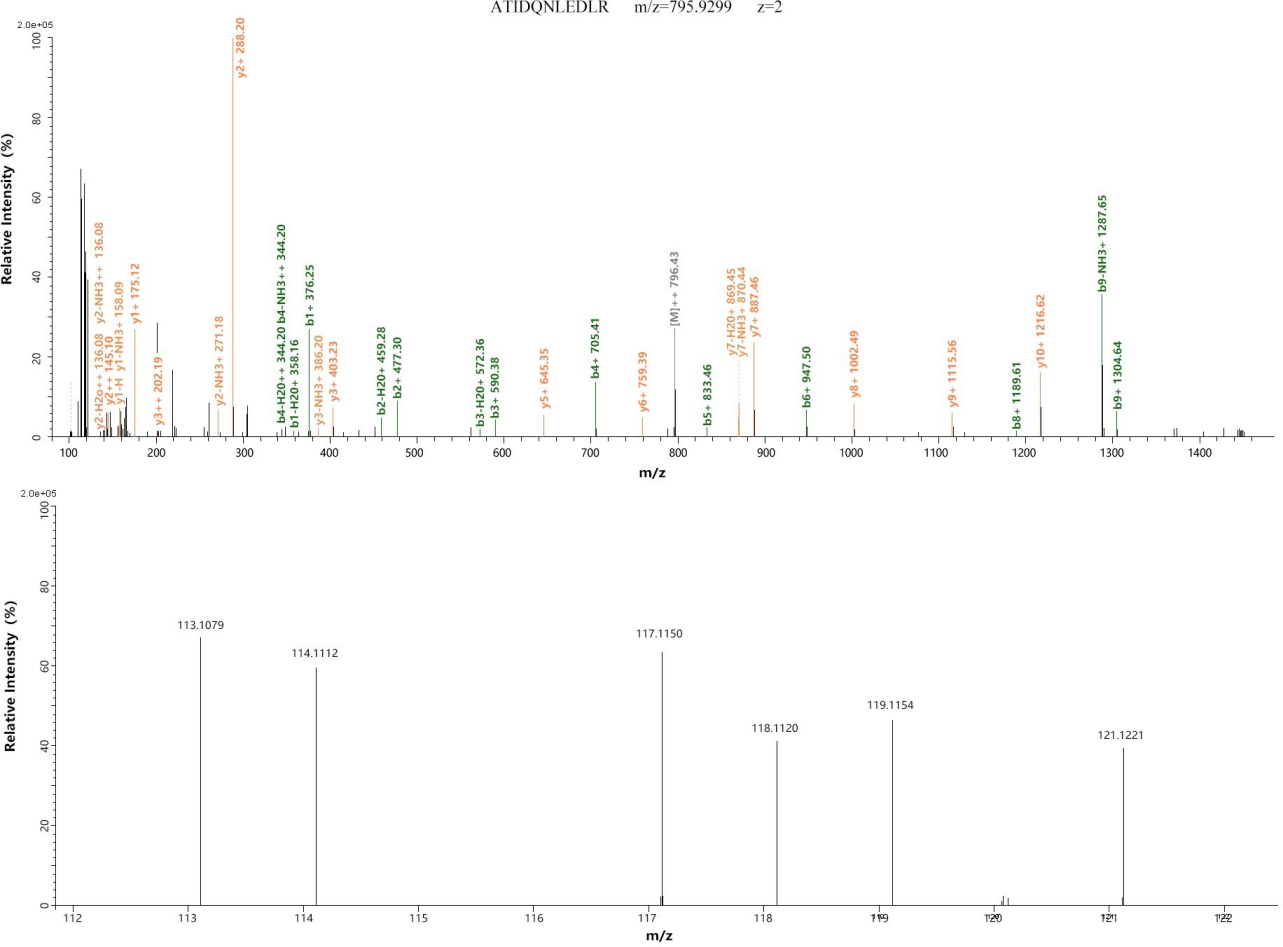
MS/MS spectra of APOA4. MS/MS spectra used for the identification and quantitation of APOA4 with 113, 114 and 117 tags for CIH samples, and 118, 119 and 121 tags for control samples. Top, the MS/MS spectra of precursor ion ATIDQNLEDLR for identifying APOA4. Bottom, relative quantitation of APOA4.

**Fig 2:**
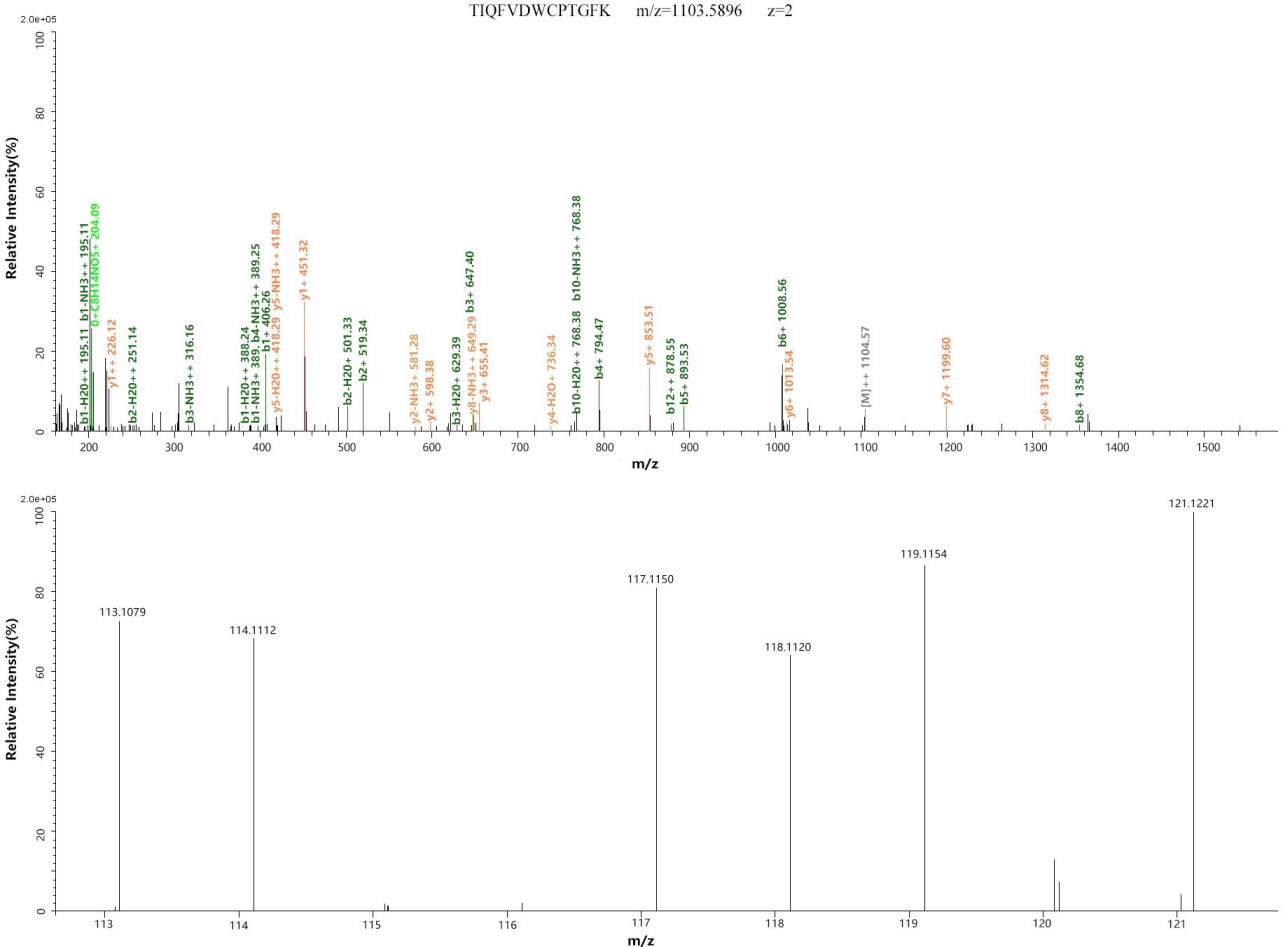
MS/MS spectra of TUBA1A. MS/MS spectra used for the identification and quantitation of TUBA1A with 113, 114 and 117 tags for CIH samples, and 118, 119 and 121 tags for control samples. Top, the MS/MS spectra of precursor ion TIQFVDWCPTGFK for identifying TUBA1A. Bottom, relative quantitation of TUBA1A.

The proteins that met the following criteria were confidently considered as DEPs: (1) proteins were identified based on ≥2 peptides; (2) proteins showed an average ratio-fold change ≥1.2, or ≤0.833 between the two analyzed groups (*t*-test, *p* <0.05). Twenty-three DEPs were identified, of which 19 were upregulated and four were downregulated. The names of these 23 DEPs and their average fold change are shown in Table 1. Among these DEPs, two proteins, APOA4 and TUBA1A, showed progressive changes after CIH, which contained two or more unique peptides and had a higher coverage. The MS/MS spectra used for the identification and quantitation of them with progressive changes are shown in Figs 1 and 2.

**Table 1:** Differentially expressed proteins identified by iTRAQ analysis.

Accession	Gene Name	Description	Average Fold Change	*p* value
Overexpressed proteins				
B1AVD2	Xpnpep2	Xaa-Pro aminopeptidase 2	1.52207	0.00284
Q9D1H9	Mfap4	Microfibril-associated glycoprotein 4	1.4335	0.00451
D3Z5G7	Ces1b	Carboxylic ester hydrolase	1.37812	0.00376
P09103	P4hb	Protein disulfide-isomerase	1.33191	0.04105
Q3U5Y3	LCP2	Lymphocyte cytosolic protein 2	1.29759	0.03305
Q8BND5	Qsox1	Sulfhydryl oxidase 1	1.29621	0.00661
Q3UWT6	Psma2	Proteasome subunit alpha type	1.27063	0.00567
Q01488	Apoa4	Apolipoprotein A-IV	1.24972	0.00332
Q8CG03	Pde5a	cGMP-specific 3’,5’-cyclic phosphodiesterase	1.24672	0.01592
A0A0G2JFZ8	Nup210l	Nuclear pore membrane glycoprotein 210-like	1.24383	0.02403
Q3T9L1	Coro1a	Coronin	1.24299	0.01998
D3YUE2	Pcolce	Procollagen C-endopeptidase enhancer 1	1.23418	7.3E-05
Q3V2X3	Il1rap	Interleukin 1 receptor accessory protein	1.2338	0.00722
Q03734	Serpina3m	Serine protease inhibitor A3M	1.23177	0.00238
D3Z450	Serpina3i	Serine (or cysteine) peptidase inhibitor, clade A, member 3I	1.22837	0.02627
Q2KHP2	Mmp19	Matrix metallopeptidase 19	1.22552	0.04392
P35918	Kdr	Vascular endothelial growth factor receptor 2	1.2214	0.0035
Q5SS40	Ywhae	14-3-3 protein epsilon	1.21811	0.03246
P29621	Serpina3c	Serine protease inhibitor	1.21766	0.01941
Underexpressed proteins				
P20826	Kitlg	Kit ligand	0.79045	0.0329
Q80U89	mKIAA0034	MKIAA0034 protein (Fragment)	0.67785	0.03657
Q6PIP8	Igh	Igh protein	0.50188	0.00236
P68369	Tuba1a	Tubulin alpha-1A chain	0.40779	0.02168

### CIH caused the upregulation of APOA4 and downregulation of TUBA1A

**Fig 3:**
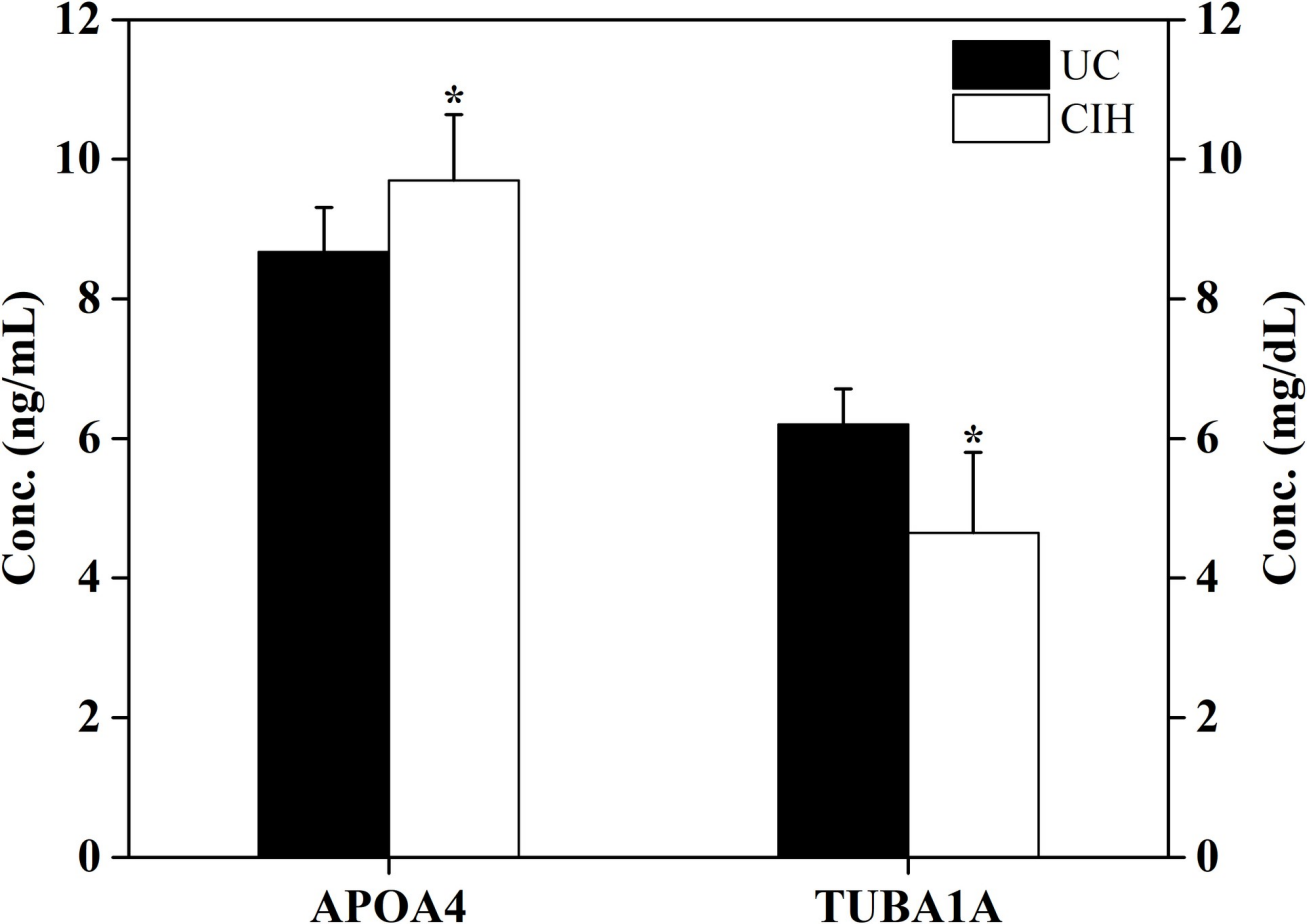
Comparison of serum APOA4 and TUBA1A levels in control and CIH groups. *p <0.05 compared with control group.

Two proteins (APOA4 and TUBA1A) with progressive changes identified by MS analysis were chosen for verification. To confirm the changes observed in the proteomic analysis, the serum concentration of two proteins was examined by ELISA in another CIH rat model (n  =  10/group). As shown in Fig 3, expression of APOA4 progressively increased, whereas expression of TUBA1A progressively decreased, consistent with the findings of the MS analysis.

### OSA caused the alteration of 2 candidate proteins

The general clinical characteristics and polysomnographic results of the 41 subjects in the control group with respect to the OSA subgroups are displayed in Table 2. No significant differences were found between the OSA and control groups regarding age (*p* = 0.208), blood pressure (*p* = 0.639 and *p* = 0.854, respectively), BMI (*p* = 0.395), and circumference of neck (*p* = 0.318) and waist (*p* = 0.119). As expected, patients with severe OSA disease had more respiratory events (AHI) and significant changes in nighttime SpO_2_ (ODI, mean SpO_2_, and lowest SpO_2_).

**Table 2:** General characteristics of each group.

Variable	Non-OSA	Mild/Moderate OSA	Severe OSA
Case (n)	12	14	15
Age (years)	36.8 ± 9.9	38.1±13.4	44.0±10.0
BMI (kg/m2)	26.4±2.2	27.2±3.7	27.8±1.8
Neck circumference (cm)	39.5±2.1	40.7±2.5	39.9±1.5
Waist circumference (cm)	93.7±6.0	98.0±10.9	100.2±5.9
AHI (events/h)	2.1±1.4	15.6±8.0^*^	57.5±10.3^*^
ODI (events/h)	2.7±1.7	18.1±10.7^*^	62.0±11.3^*^
L-SpO2 (%)	87.4±3.8	77.8±9.3^*^	62.4±7.0^*^
M-SpO2 (%)	94.6±0.9	93.4±3.5^*^	89.7±1.5^*^
SBP (mmHg)	131.6±14.7	133.9±11.1	136.5±13.9
DBP (mmHg)	88.9±12.1	86.7±9.2	88.2±9.6

Data are presented as median ± SD.BMI: Body mass index, AHI: Apnea/hypopnea index, ODI: Oxygen desaturation index, L-SpO2: Lowest blood oxygen saturation during recording time, M-SpO2: Mean blood oxygen saturation during recording time, SBP: Systolic blood pressure, DBP: Diastolic blood pressure.

**Fig 4:**
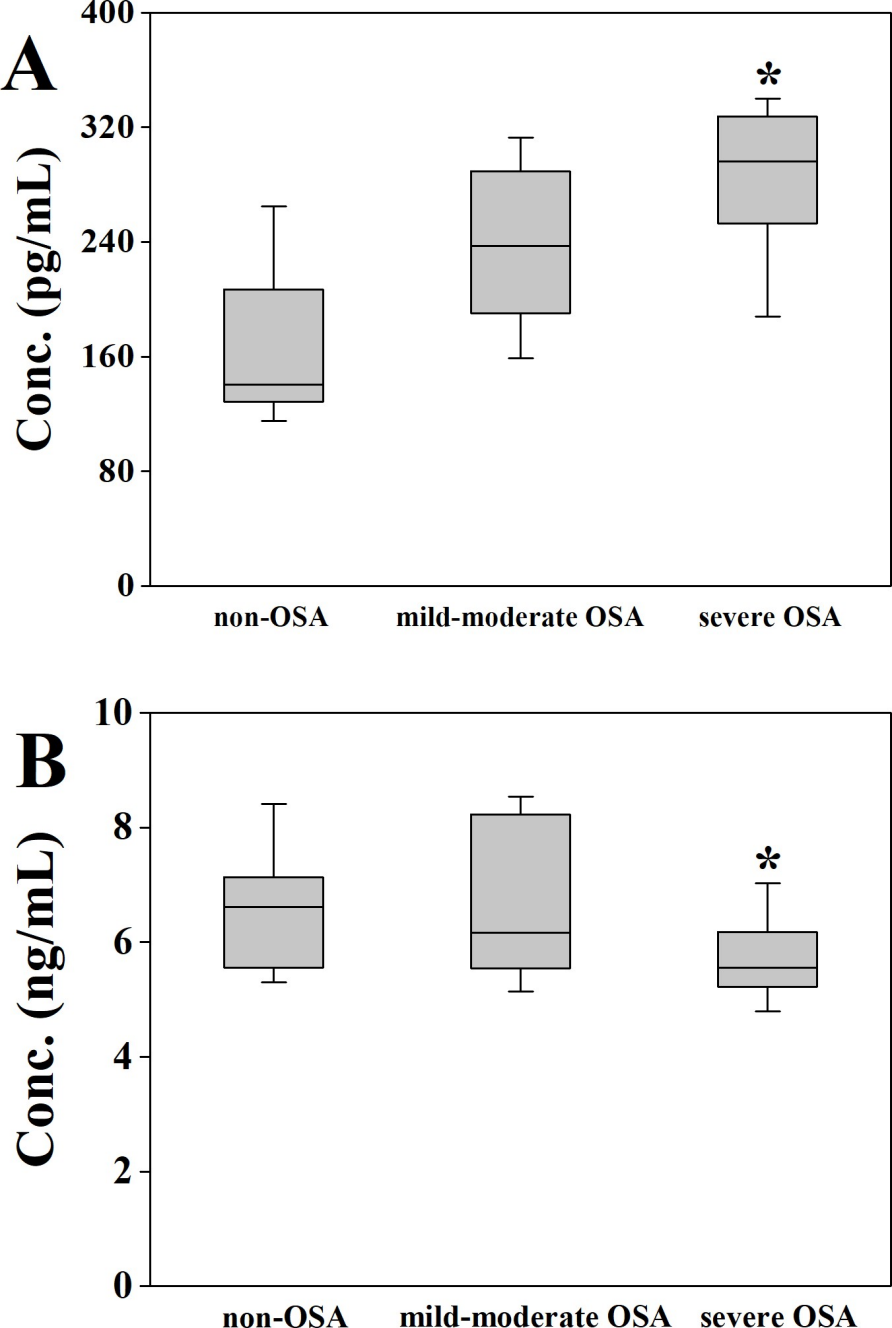
Expression of APOA4 and TUBA1A in three OSA subgroups. *p <0.05.

Clinical studies demonstrated that patients in the severe OSA group showed higher levels of APOA4 (285.84±47.97 pg/mL) than those with non-OSA or mild-moderate OSA (163.03±51.25 pg/mL and 237.32±49.80 pg/mL, *p* <0.01, and *p*  = 0.013, respectively; Fig 4A). Compared to severe OSA (5.72±0.74 ng/mL), TUBA1A level elevated significantly in control and mild-moderate OSA group (6.49±0.99 ng/mL and 6.68±1.29 ng/mL, *p* = 0.028 and *p* = 0.037, respectively, Fig 4B).

**Fig 5:**
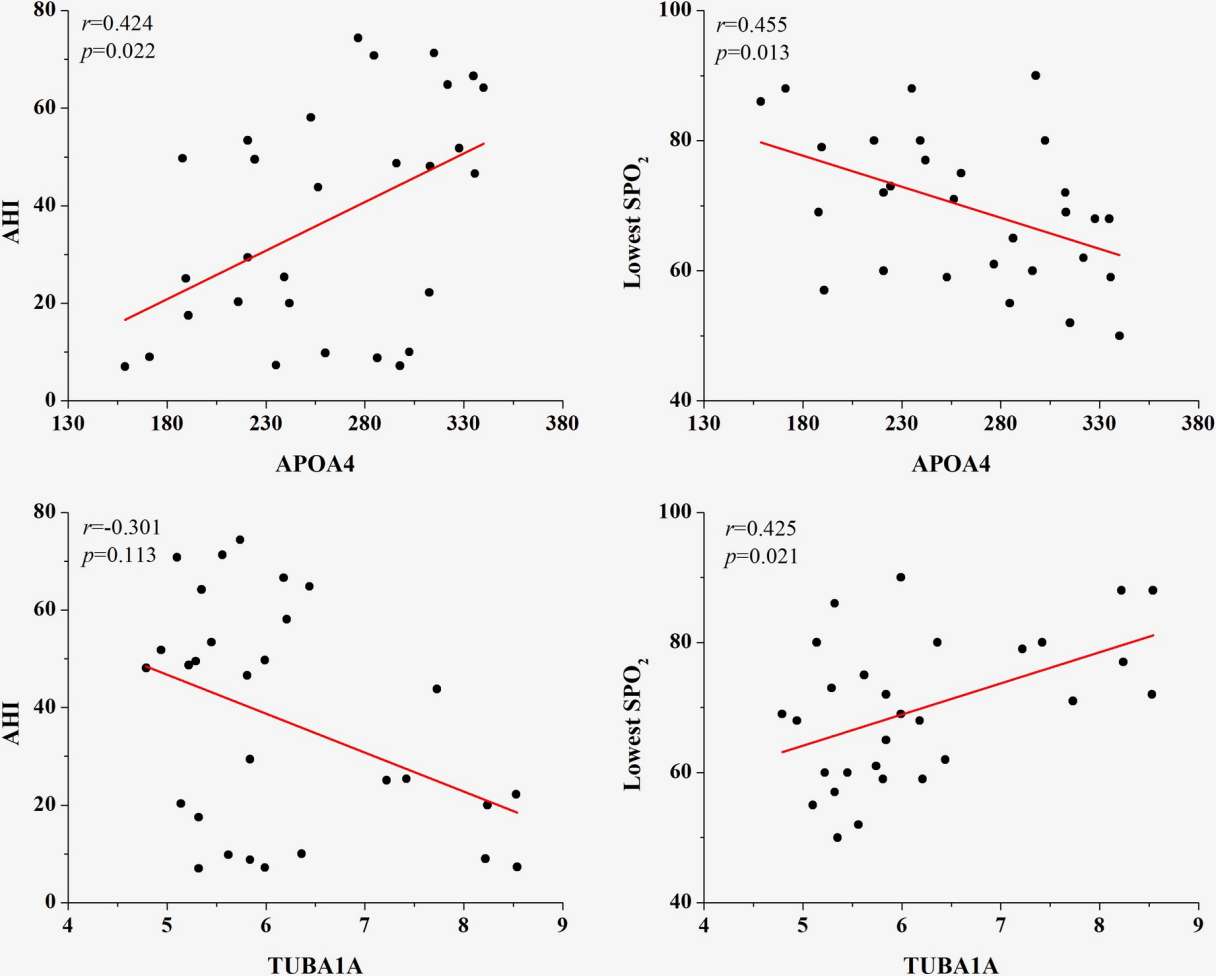
The relation between four differentially expressed proteins and polysomnography parameters.

Further statistical analyses were performed using the Spearman correlation test to analyze the correlations between the two protein levels in the patients’ serum with AHI and lowest SpO_2_. APOA4 concentration was inversely correlated with AHI and negatively correlated with lowest SpO2 (Fig 5). TUBA1A levels were positively correlated with lowest SpO_2_, but no significant correlation was observed between TUBA1A and AHI (*p*  = 0.113).

### Hierarchical clustering, gene-ontology and KEGG pathways analysis of the differential proteins

**Fig 6:**
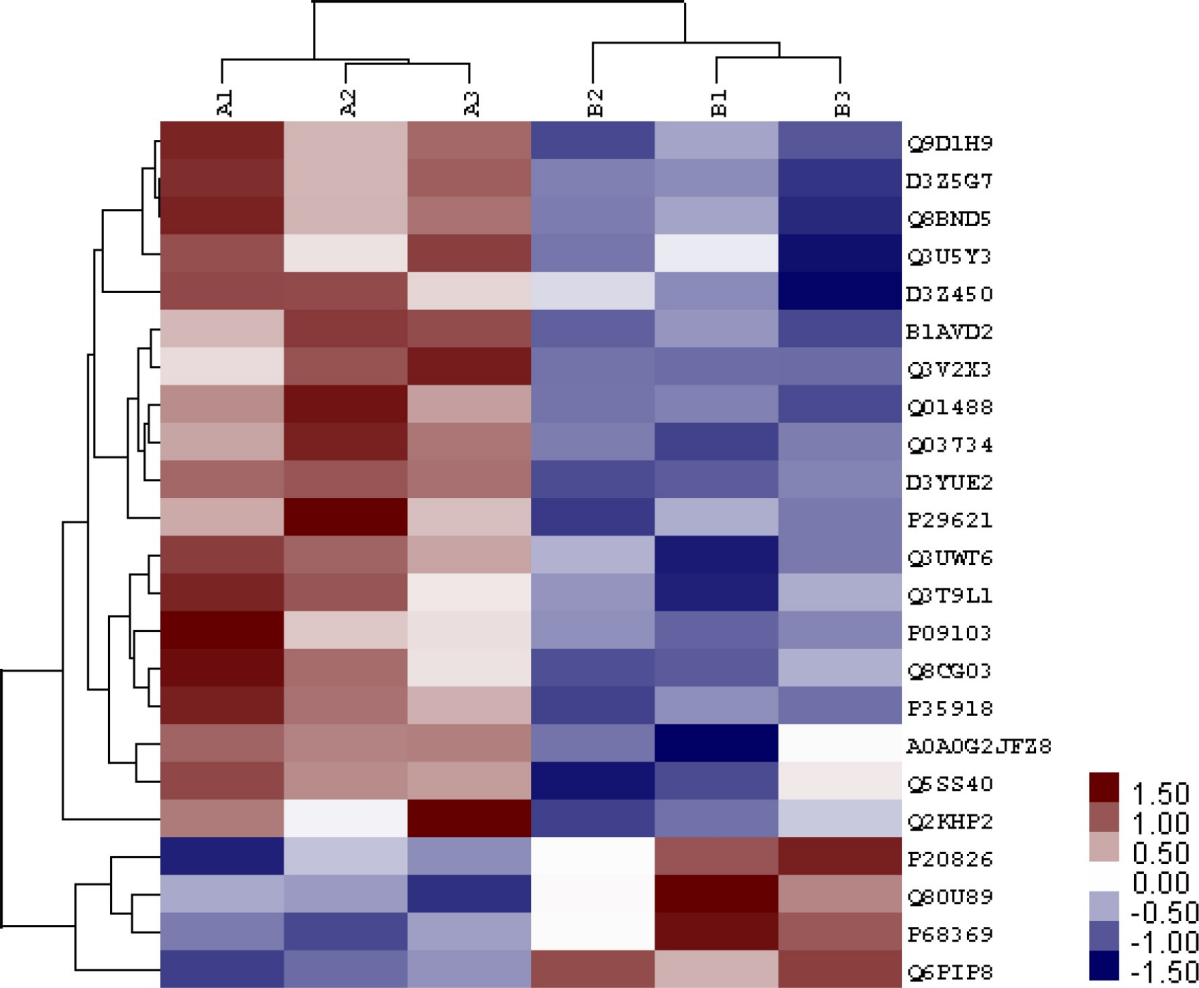
Hierarchical clustering of 23 differentially expressed proteins. Log2 of normalized counts of differential proteins were clustered. Red clusters denote high levels of expression whereas blue clusters denote low levels of expression. The x axis represents the samples whereas the y axis represents the proteins.

**Fig 7:**
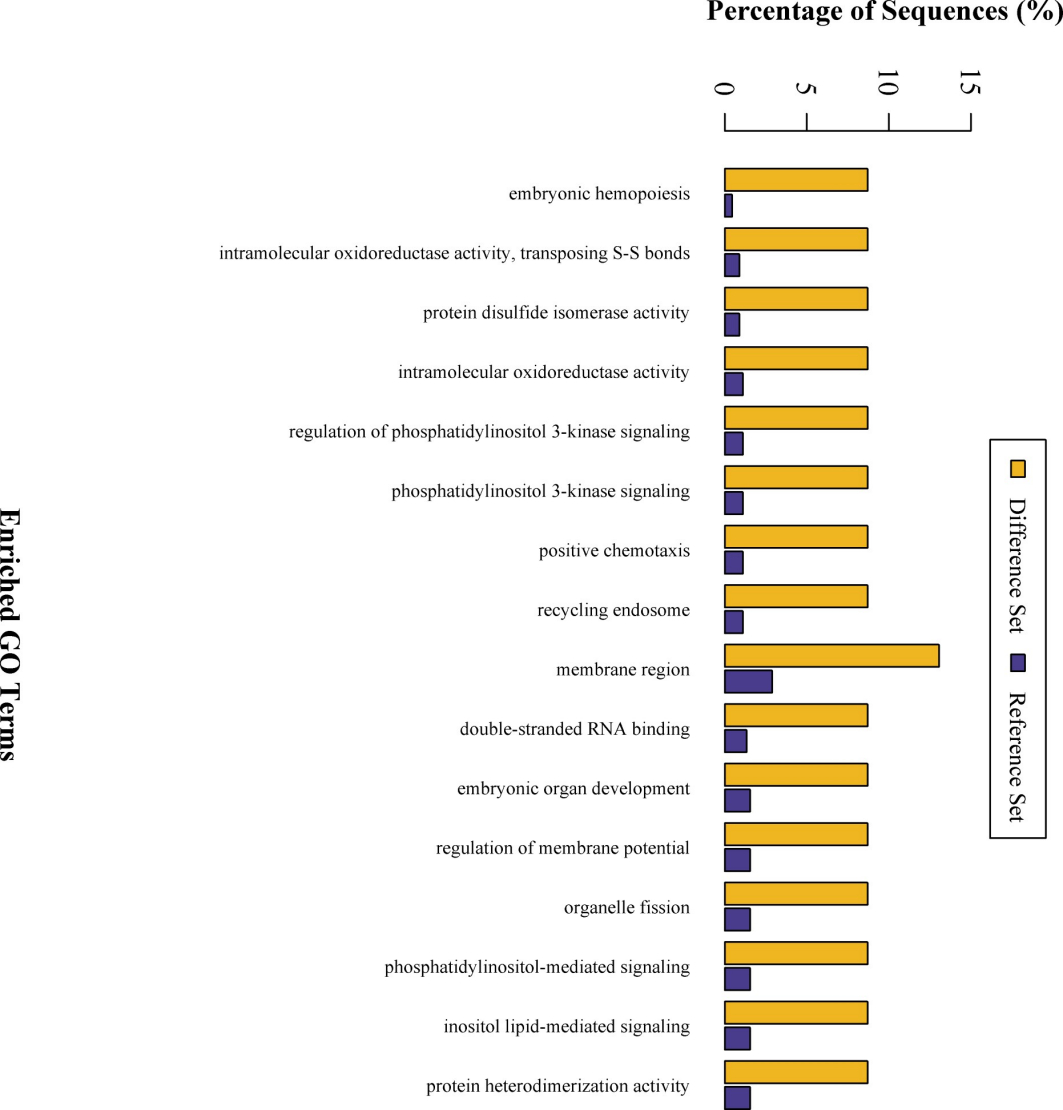
GO enrichment analysis of differential expression proteins. The x axis represents the enrichment GO function whereas the y axis represents the sequence percentage. The yellow bar represents the differential set and the blue bar represents the reference set.

**Fig 8:**
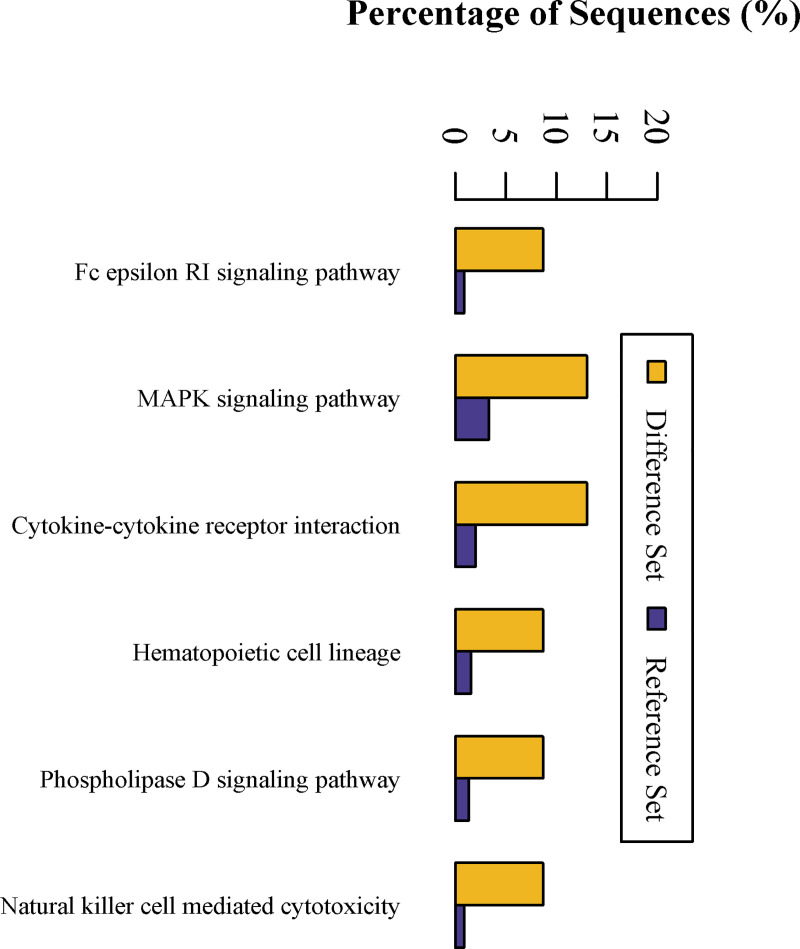
KEGG enrichment analysis of differential expression proteins. The x axis represents the pathway in which the different proteins participate, and the y axis represents the sequence percentage. The yellow bar represents the differential set and the blue bar represents the reference set.

To gain more insight into the biological significance of the DEPs in the CIH process, hierarchical clustering was performed on 23 DEPs. All DEPs were hierarchically divided into four groups (Fig 6). Proteins within the same cluster were coregulated proteins that might have similar biological functions during bronchial epithelial carcinogenesis. GO analysis revealed that DEPs are enriched with proteins of different functions and may play a distinctive role during the CIH process (Fig 7). KEGG pathway analysis showed that DEPs are involved in signaling pathways, such as the mitogen-activated protein kinase (MAPK) signaling pathway and cytokine-cytokine receptor interaction, and are involved in immunology or metabolism-associated signaling pathways, such as the Fc epsilon RI signaling pathway, natural killer cell mediated cytotoxicity, phospholipase D signaling pathway, and hematopoietic cell lineage (Fig 8). DEPs may play a role in CIH via these signaling pathways.

## Discussion

Irrespective of rhinitis or other causes of OSA [16], CIH is treated as the main pathological process and a potential factor of OSA complications. The direct consequence of CIH is oxidative stress and inflammation, which are linked to disease evolution and comorbidities [17]. It is important to identify proteins with altered expression during CIH to determine the functional mechanisms and predictors for better OSA treatment as well as to assess the severity and prognosis of OSA. Several recent proteomics studies have aimed to elucidate the pathophysiology of OSA. However, no study has fully explained the effect of OSA, especially CIH, on proteomics. In addition, there are many confounding risk factors in human research that cannot completely rule out whether the subjects have other OSA-induced diseases [10]. Thus, it is critical to explore the specific pathophysiology and underlying mechanisms of CIH in OSA.

In our study, iTRAQ labeling was performed to identify differential serum proteins in CIH. In the present study, we found that on CIH intervention, the serum proteome of rats changed. A total of 23 differential proteins were identified, and four differential proteins (APOA4, IL1RAP, CORO1A, and TUBA1A) were selectively validated in the rebuilt-CIH model of rats and patients with OSA. Next, we evaluated the expression levels of four differential proteins (APOA4, IL1RAP, CORO1A, and TUBA1A) and found that the upregulation of APOA4 and downregulation of TUBA1A were consistent among the three methods.

APOA4 is synthesized and secreted by the intestine. The physiological role of APOA4 has not yet been fully elucidated. It is not only found to have anti-atherosclerosis, anti-oxidant, anti-platelet, and anti-inflammatory properties but is also related to reverse cholesterol transport and plasma glucose metabolism. Many studies have found that low APOA4 levels are associated with arteriosclerotic cardiovascular disease [18–20]. Elevated APOA4 levels can prevent atherosclerotic cardiovascular diseases and improve glucose homeostasis. In anti-atherosclerotic effects, upregulation of APOA4 has the ability to mediate reverse cholesterol transport and reduce inflammatory and oxidative stress. In plasma glucose metabolism effects, APOA4 confers higher insulin secretion to decrease elevated plasma glucose levels [21]. A recent study has shown that APOA4 is a useful marker for the level of triglycerides in the body after fat intake, indicating that APOA4 is positively correlated with triglycerides during a fat-rich diet and fasting. APOA4 improves plaque stability in rats by enhancing anti-inflammation of plaque, anti-oxidative and anti-apoptosis, and directly downregulating mmp-9 gene and protein expression in macrophages [22]. In mice, the anti-inflammatory properties of APOA4 are reflected by the low levels of pro-inflammatory cytokines and atherosclerotic lesions [23].

In summary, APOA4 can protect against lipid peroxidation, inhibit the progression of atherosclerosis, and enhance insulin secretion in OSA patients. The present study revealed that APOA4 increased during CIH and OSA patients without atherosclerotic cardiovascular disease and diabetes mellitus and may be a potential marker for OSA complications. Therefore, it is conceivable that in patients with OSA, without complications, APOA4 levels are elevated under CIH stimulation and provide protection but with progression of OSA, the lower the APOA4 level, the higher the incidence of complications.

Tubulin alpha-1a chain (TUBA1A) is a major component of adult α-tubulin mRNA and is involved in the formation of microtubules. Microtubules are the construction of cytoskeleton, cilia, flagella, axon fibers, and mitotic spindles that play key roles in numerous essential functions, including axon and dendrite growth and neuron migration throughout the brain [24]. The *TUBA1A* gene is highly conserved among various species. During embryonic development, TUBA1A constitutes up to 95% of all α-tubulin mRNAs in the brain [25]. TUBA1A expression is dramatically reduced in adulthood; however, some regions, including the brain (hippocampus, cerebellum), olfactory neurons, and lateral sensory cells, retain TUBA1A expression. TUBA1A proteins may participate in microtubules during neurodevelopment and remain within the microtubule network of adult neurons [25–30]. Previous studies on TUBA1A have focused on anencephaly during neocortex development caused by mutations in the *TUBA1A* gene [31]. In recent years, a study found that TUBA1A is required for adult neuronal function and proper functioning throughout their lifetime. Downregulation of TUBA1A has been found to be associated with reduced microtubule tracks in neurites and late-onset behavioral deficits [32].

In addition, TUBA1A was associated with sperm motility. Many protists and metazoan sperm cannot move without microtubules, because the complex of microtubules and motors is the core of cilia and flagella [33]. Eleven microtubules, including TUBA1A, are components of the sperm tail [34]. The study found that the K40 site-reversible tubulin acetylation site can specifically regulate microtubule stability in sperm [35].

In general, TUBA1A is a microtubule that mediates axonal transport, dendritic transport, and sperm motility. In the present study, we found that downregulation of TUBA1A in CIH rats and patients with OSA may be an underlying mechanism for OSA complications, such as cognitive disorders and male hypogonadism. Thus, on one hand, CIH and OSA may downregulate TUBA1A, resulting in the reduction of microtubule formation and obstruction of intracellular transport of neurons, leading to synaptic dysfunction and cognitive dysfunction; on the other hand, the reduction of TUBA1A reduces the regulation of reversible lysosase-acetylation and affects the construction of axonal microtubules, leading to insufficient sperm motility and male hypogonadism.

In cellular experiments, it was found that NO cannot induce hypoxia-inducible factor-1 activation without the MAPK signaling pathway [36]. In an animal model, CIH was found to activate the MAPK signaling pathways, which disturbed insulin secretion and led to pancreatic inflammation [37], liver fibrosis [38], and brain injury [39]. Experiments on whole-exome sequencing and circulating microRNAs have shown that cytokine-cytokine receptor interactions are associated with Alzheimer’s disease in patients with OSA [40] and endothelial dysfunction [41]. Although genomics or proteomics results about CIH or OSA differ from ours, there are several common proteins, pathways, and functional proteins. Analysis of long non-coding RNA expression in the rat model of CIH showed that cytokine-cytokine receptor interactions were enriched in downregulated transcripts [11]. According to Jurado-Gamez, Bernabe et al. [10] and Li et al. [42], a serum proteomic study showed that APOA4 expression changed in OSA patients and CIH rat models, which are the same as ours. This suggests that the MAPK signaling pathway and cytokine-cytokine receptor interaction play a vital role in CIH.

There are limitations due to the small sample size of the rats used in the experiment, the number of differential proteins was too less to form the protein interaction network diagram, and CIH intervention was conducted during the day when the rats were not in the sleep state, which was insufficient to simulate the real state of intermittent hypoxia in OSA patients. The present proteomics study demonstrated that four proteins were differentially expressed in the serum of CIH rats. Of these proteins, APOA4 and TUBA1A were verified by ELISA in rats and 41 subjects. Furthermore, APOA4 was positively correlated with AHI, and both APOA4 and TUBA1A were correlated with the lowest SpO2. Further studies will focus on exploring changes in the two identified proteins (APOA4 and TUBA1A) after treatment with OSA.

## Conclusion

The use of iTRAQ-labeling combined with two dimensional LC-MS/MS identified 23 DEPs during the CIH process, and the serum protein expression profile of CIH rats was successfully mapped. Two DEPs (APOA4 and TUBA1A) with progressive changes were verified. We found that APOA4 and TUBA1A had the same expression pattern in the three experiments and may serve as novel potential biomarkers for complications of OSA, such as cardiovascular disease, cognitive dysfunction, and male hypogonadism. The findings reported here could have potential clinical value in predicting OSA complications and provide valuable information for further study of molecular mechanisms.

## Supporting information

S1 File Study data. (XLSX)
